# Fabrication of binary metal–organic frameworks of Ni–Mn@ZIFs(Co_x_·Zn_1−x_O) decorated on CF/CuO nanowire for high-performance electrochemical pseudocapacitors

**DOI:** 10.1038/s41598-024-64307-x

**Published:** 2024-06-12

**Authors:** Ali Momeni Abkharaki, Ali A. Ensafi

**Affiliations:** 1 Department of Chemistry, Isfahan University of Technology, Isfahan, 84156-83111 Iran; 2 Department of Chemistry & Biochemistry, University of Arkansas, Fayetteville, AR 72701 USA

**Keywords:** Renewable energy, Pseudocapacitors, Binary MOF, ZIFs, Synergistic performance, Chemistry, Energy science and technology, Materials science, Nanoscience and technology

## Abstract

Herein, metal–organic frameworks (MOFs) derived nanoflower-like based binary transition metal (Ni–Mn) are successfully fabricated by a simple synthesis method. The fabricated nanoflower-like structure displays a unique nanoflower-like architecture and internal porous channels constructed by MOF coated on CuO/CF/ZIFs (Co_x_·Zn_1−x_O) substrate, which is beneficial for the penetration of electrolyte and electron/ion transportation. The as-prepared CF/CuO/ZIFs (Co_x_·Zn_1−x_O)@BMOF(Ni–Mn) electrode materials present significant synergy among transition metal ions, contributing to enhanced electrochemical performances. The as-prepared CF/CuO/ZIFs (Co_x_·Zn_1−x_O)@BMOF(Ni–Mn) hybrid nanoflower-like display a high specific capacity of 1249.99 C g^−1^ at 1 A g^−1^ and the specific capacitance retention is about 91.74% after 5000 cycles. In addition, the as-assembled CF/CuO/ZIFs (Co_x_·Zn_1−x_O)@BMOF(Ni–Mn)//AC asymmetric supercapacitor (ASC) device exhibited a maximum energy density of 21.77 Wh·kg^−1^ at a power density of 799 W kg^−1^, and the capacity retention rate after 5000 charge and discharge cycles was 88.52%.

## Introduction

The pressing energy issues resulting from the overconsumption of fossil fuels and the detrimental repercussions of their burning, such as air pollution and greenhouse gas emissions, are the primary drivers for investigating the progress of alternative energy sources^1,2^. The International Energy Agency (IEA) reports that around 80% of our energy demands are fulfilled by using fossil fuels^3^. The scientific investigation of renewable energy sources and energy storage devices has increased significantly due to the depletion of fossil fuels and the advancement of the global economy. The tremendous advancements in materials technology have resulted in a growing demand and widespread utilization of high-performance energy storage devices^4^. These devices are utilized in several industries including portable electronics (smartphones, smartwatches, and laptops), electric cars, and so on^5,6^. Supercapacitors (SCs) possess distinct electrochemical qualities that give them an advantage over batteries and physical dielectric capacitors. These properties include high power density, long life span, environment-friendliness, high specific capacity, and short charging time. The operation mechanism of the SCs depends on either the separation of charges in the Helmholtz double-layer (electrical double-layer capacitor), or the Faradaic redox reaction (pseudo-capacitor)^7,8^.

Researchers have extensively studied CuO nanoparticles with different morphology due to their enhanced optical, electrical, and surface properties^9^. Currently, a significant amount of research is focused on studying the surface chemistry of CuO nanostructures that have been modified with several materials. The structural architecture of CuO can be manipulated at the nanoscale by modifying their dimensions or morphologies, such as nanowires, spherical, and so on^10–12^. The energy storage properties of CuO are significantly impacted by both electrical properties and active surface sites, which play a crucial role in SCs and contribute to the optimal characteristics of SCs^13,14^. Typically, CuO is converted to its metallic form during charging and then returned to its original state by discharge. These reversible redox reactions facilitate the effective storage and discharge of electrical energy, hence contributing to the elevated capacitance of supercapacitors^15–17^. The transition metal hydroxide or oxide materials, such as Co (OH)_2_, Ni (OH)_2_, Co_2_O_3_, and NiO, are commonly employed in merged with nanostructure to enhance the mechanical characteristics and surface area of the electrode^18,19^. The semiconductor such as ZnO and TiO_2_ have been doped with various transition metals such as Co, Mn, Fe, and Ni and have been extensively studied^20,21^. This is because theoretical predictions suggest that these zinc when substituted for transition metals in the wurtzite lattice, exhibit a higher degree of solid solubility despite having similar valence states. Calcination of the metal–organic framework (MOF) is an effective method for concurrently integrating metals into the structure of compounds.

MOF can combine the positive qualities of separate components, resulting in synergistic effects that yield a greater variety of structures, morphologies, and properties compared to using single ingredients alone^22^. The adjustable structural arrangement and controlled composition of MOFs provide numerous advantages in electrochemical energy storage applications, including (A) Enabling the efficient transport of electrolyte ions through the pores. (B) The network has easily accessible metal nodes that can undergo redox reactions, and these nodes are located at the inorganic building blocks of the network. (C) Customizing the architecture of MOF structure to produce specific shape-selective effects^23,24^. Guiwu Liu, Haohua Li et al. employed porous TiO_2_ nanotablets with abundant oxygen vacancies were produced through the thermal decomposition of MIL-125^25^. Various investigations have been conducted on diverse monometallic and bimetallic MOF materials, focusing on areas such as catalytic activity, energy storage systems, energy conversion, and more. In addition, the solvothermal and hydrothermal technique was employed to fabricate monometallic and bimetallic MOF using selected transition metals. Zeolitic imidazolate frameworks (ZIFs) are a kind of MOF that are made by coordinating metals (such as Co, Zn, Ni, Mn, and so on) with imidazolate-type linkers^26^. Every metal cation results in a unique kind of ZIF, which displays the distinctive features of both the metal and framework. Currently, there have been over 150 documented structures of ZIFs^27^. Ahmad et al. employed ZIF-67 to synthesize nitrogen-doped carbon nanotubes (CNTs) and subsequently modified them with sulfur and Ni (OH)_2_. These modified CNTs were then evaluated as electrode materials with high potential for supercapacitors^28^. These frameworks have shown significant potential for fabricating porous materials with unique morphology. The porous materials generated from zinc-based ZIF-8 or cobalt-based ZIF-67 exhibit numerous favorable characteristics, as well as certain limits^29–31^. So, it is preferable to incorporate the properties of zinc and cobalt ions within a single crystal (known as bimetallic ZIF) to develop porous materials that possess customized functionalities^32^. The bimetallic ZIFs (Co_x_·Zn_1−x_O) compound has lattice and band structure characteristics that can enhance the performance of supercapacitors. Furthermore, ZIFs have the potential to be combined with various nanomaterials, including transition metal oxides, sulfides, and hydroxides, in order to greatly augment their energy storage capacities^33,34^. Hybrid crystalline porous materials (HCPM) are an excellent choice for achieving the specifications of advanced energy conversion and storage technologies^35,36^. The binary MOF exhibited superior characteristics such as a large surface area, well-defined crystal structure, improved redox properties, and exceptional stability when compared to the monometallic MOF. This can refer to the binary metal–organic framework (BMOF) that exhibits numerous oxidation states in transition metals. This characteristic allows for an improved faradic redox reaction and higher electrical conductivity, which ultimately benefits the performance of SCs^37,38^. The binary MOF is synthesized by the hydrothermal method using self-assembled hexamine coordination frameworks (BMOF), with transition metal components.

The present study focuses on investigating the electrochemical characteristics of HCPM composed of BMOF (Ni–Mn), Co_x_·Zn_1−x_ structure, and CuO layer to enhance the performance of supercapacitors. The CuO nanowire and ZIFs (Co_x_·Zn_1−x_O), substrates were deposited on copper foam (CF) via simple hydrothermal steps, respectively. The BMOF structures, composed of Mn and Ni, were synthesized utilizing a hydrothermal approach on the specified layer. The CF/CuO/ZIFs (Co_x_·Zn_1−x_O)@BMOF(Ni–Mn) electrode, which was fabricated without the use of a binder, had exceptional capacitive performance in SCs. The morphological and electrochemical characteristics of all electrode layers have been extensively investigated. Integrating BMOF (Ni–Mn) with a ZIFs Co_x_·Zn_1−x_O, substrate can greatly enhance the capacity and energy density in comparison to other layers. The CF/CuO/ZIFs (Co_x_·Zn_1−x_O)@BMOF(Ni–Mn) electrode demonstrated exceptional capacity retention and a low charge-transfer resistance (Rct) at the electrode–electrolyte interface.

## Results and discussion

### Characterization

**Figure 1:**
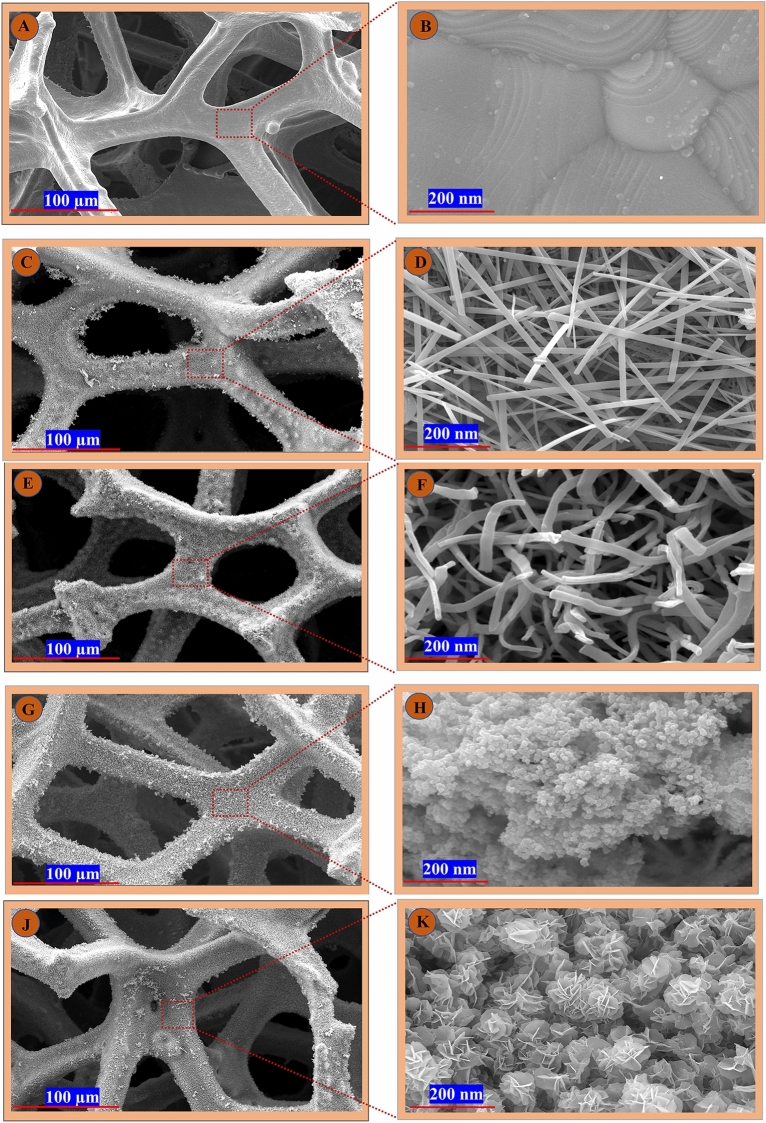
FESEM micrographs of (**A,B**) Commercial CF images, (**C,D**) CF/Cu(OH)_2_ images, (**E,F**) CF/CuO images, (**G,H**) CF/CuO/ZIFs (Co_x_·Zn_1−x_O) images, (**J,K**) CF/CuO/ZIFs (Co_x_·Zn_1−x_O)@BMOF(Ni–Mn) images.

Porosity and high surface area are two fundamental factors that extensively determine the effectiveness and efficiency of nanostructures in energy storage applications. The as-prepared electrodes were examined with FESEM analysis to investigate the morphology, sizes, and distribution of grains. As can be seen in Fig. 1A–J, introducing elemental components can induce morphological alterations in an electrode surface, which can either augment or reduce its characteristics. The FESEM of commercial CF exhibits a smooth surface devoid of any pores, as expected (shown in Fig. 1A,B). The study presents Cu (OH)_2_ and CuO fabricated on CF substrates that were synthesized using different approaches. As shown in Fig. 1C,D, the high-magnification FESEM image of Cu (OH)_2_ confirms the structure of nanowires, characterized by a substantial length-to-diameter ratio. Under the calcination step, the CuO morphology displays the formation of multi-branched nanowires and nanorod-type structures (Fig. 1E,F). The porous surface of the CuO layer allows for an open structure in supercapacitor electrodes, which promotes fast ion diffusion at the interface between the electrolyte and the electrode. The incorporation of ZIFs (Co_x_·Zn_1−x_O) into the CuO substrate changed its morphology. As shown in Fig. 1G,H. The addition of ZIFs (Co_x_·Zn_1−x_O) increased the agglomeration of spherical morphology, resulting in the formation of a uniformly structured material with high porosity. The high surface area of CF/CuO/ZIFs (Co_x_·Zn_1−x_O) also offers a greater number of electro-active sites, as well as a wider contact area between the electrolyte and electrode. This results in a higher charge–discharge capacity of supercapacitors. The high-resolution image of CF/CuO/ZIFs (Co_x_·Zn_1−x_O)@BMOF(Ni–Mn) exhibited a nanoflower-like structure, Fig. 1I,J. Among the electrodes with different structures, nanoflower-like structures of CF/CuO/ZIFs (Co_x_·Zn_1−x_O)@BMOF(Ni–Mn) possess numerous advantages among the other electrode morphologies. Due to their shorter paths for ion and electron transport, high surface area, and structural stress reduction across consecutive electrochemical cycles. The result related to the morphology of electrodes and active sites has been deeply investigated by different electrochemical methods.

**Figure 2:**
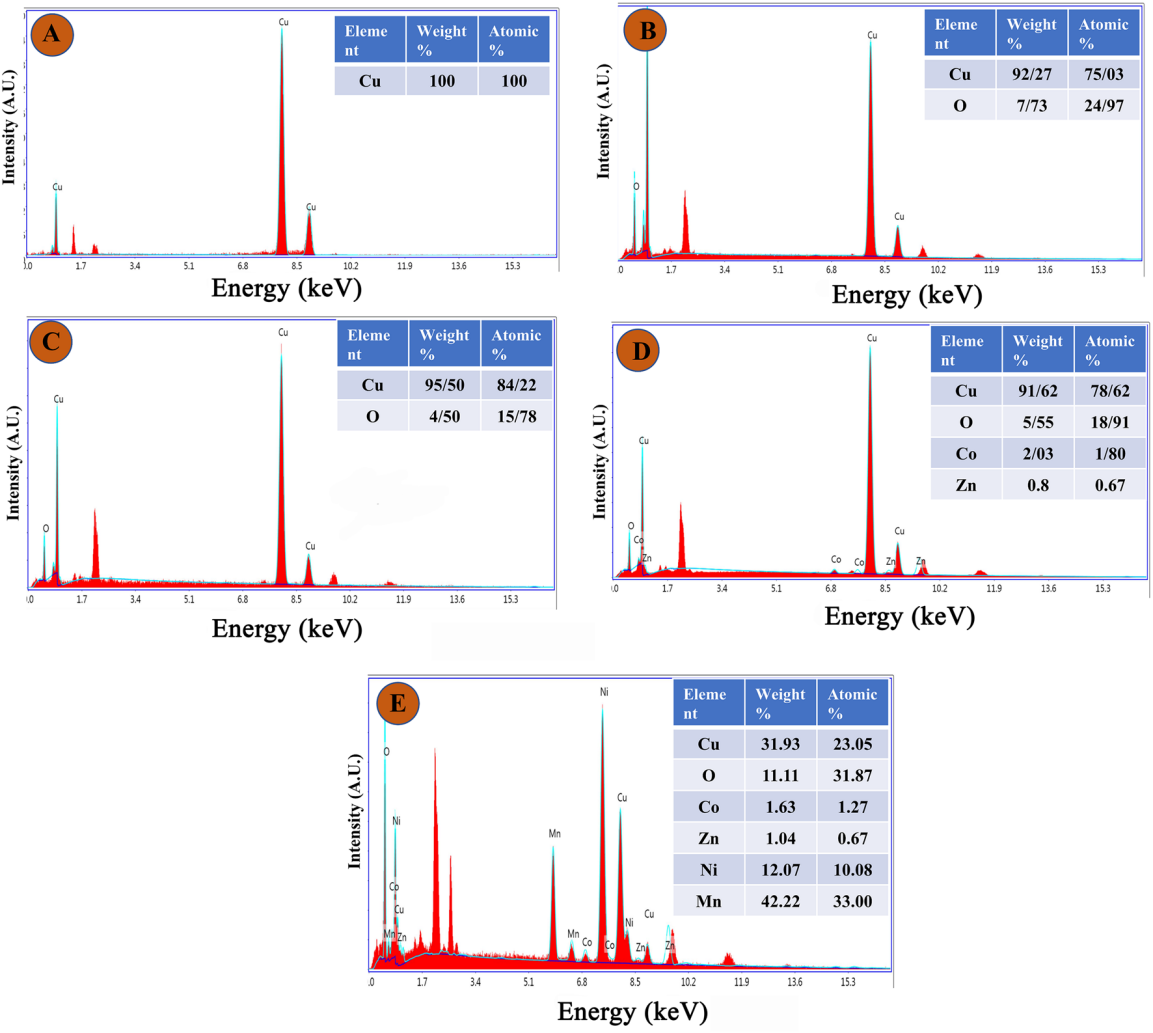
EDS spectra and elemental table ratio of (**A**) Commercial CF, (**B**) CF/Cu(OH)_2_, (**C**) CF/CuO, (**D**) CF/CuO/ZIFs (Co_x_·Zn_1−x_O), (**E**), CF/CuO/ZIFs (Co_x_·Zn_1−x_O)@BMOF(Ni–Mn).

**Figure 3:**
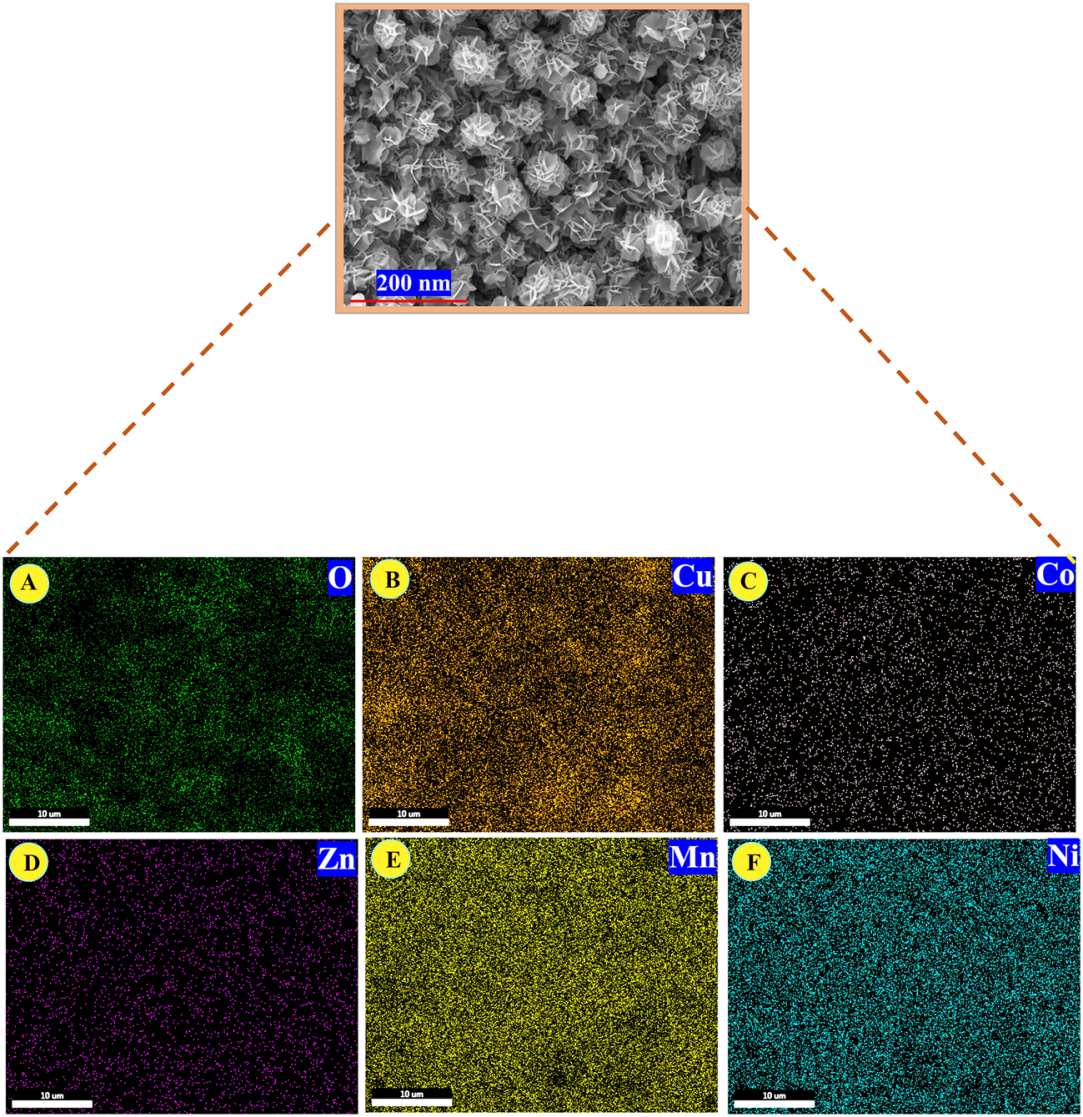
Elemental mapping of elements present in CF/CuO/ZIFs (Co_x_·Zn_1−x_O)@BMOF(Ni–Mn). (**A**) Oxygen, (**B**) Copper, (**C**) Cobalt, (**D**) Zinc, (**E**) Manganese, (**F**) Nickel.

The elemental compositions of the electrode materials were analyzed using energy dispersive spectroscopy (EDS). Figure 2A–E displays typical EDS spectra for CF/Cu(OH)_2_, CF/CuO, CF/CuO/ZIFs (Co_x_·Zn_1−x_O), and CF/CuO/ZIFs (Co_x_·Zn_1−x_O)@BMOF(Ni–Mn) respectively. The atomic ratio table of the different elements exhibited inside the spectra. The presence of all materials is confirmed by the elemental ratio and EDX signal of the CF/CuO/ZIFs (Co_x_·Zn_1−x_O)@BMOF(Ni–Mn) electrode. Elemental mapping of components found in CF/CuO/ZIFs (Co_x_·Zn_1−x_O)@BMOF(Ni–Mn) electrode is illustrated in Fig. 3A–F.

**Figure 4:**
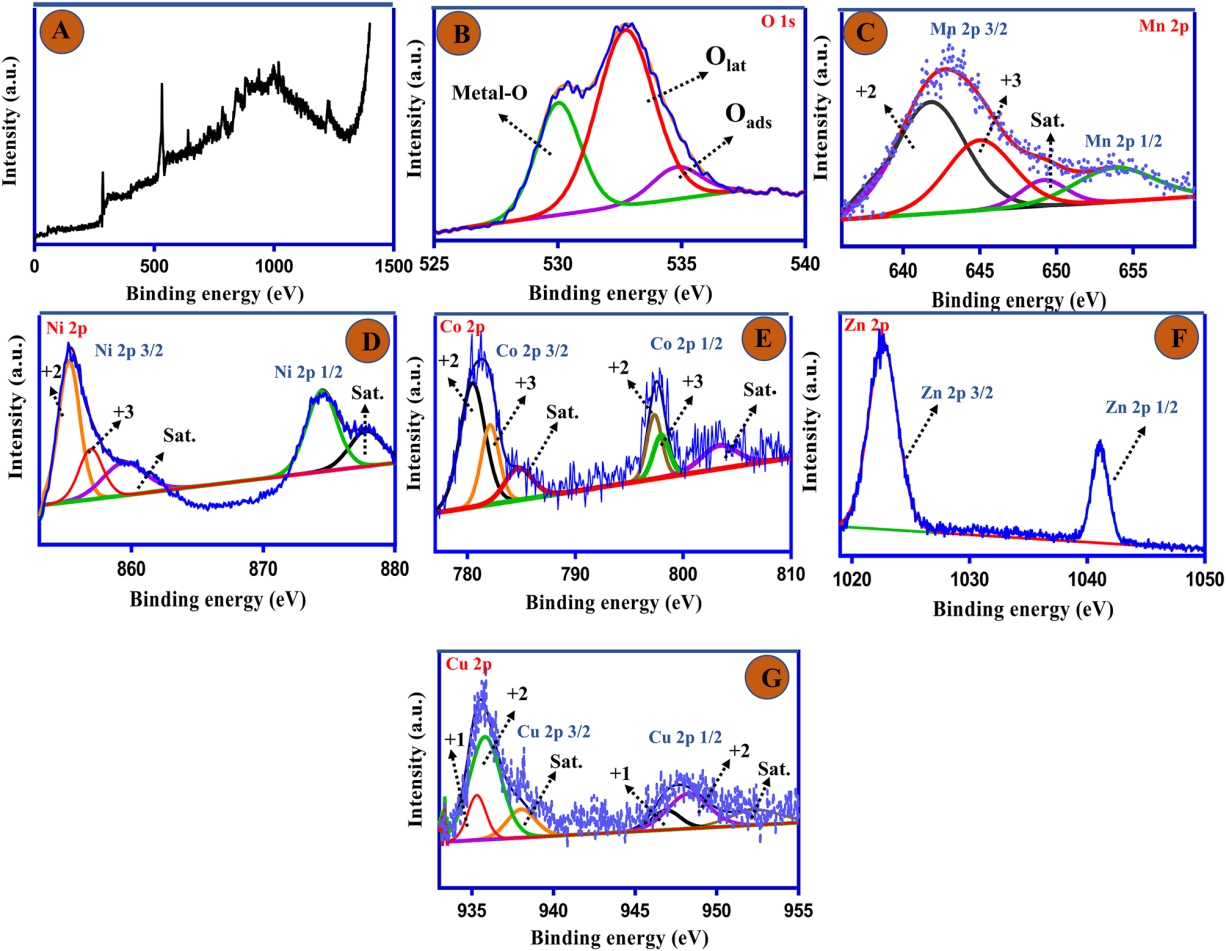
XPS spectra of CF/CuO/ZIFs (Co_x_·Zn_1−x_O)@BMOF(Ni–Mn) electrode. (**A**) survey scan spectra. Core-level XPS spectra of, (**B**) O 1s, (**C**) Mn 2p, (**D**) Ni 2p, (**E**) Co 2p, (**F**) Zn 2p, (**G**) Cu 2p.

The chemical states and elemental compositions of the CF/CuO/ZIFs (Co_x_·Zn_1−x_O)@BMOF(Ni–Mn) electrode were determined through the utilization of X-ray Photoelectron Spectroscopy (XPS). For the XPS analysis, the binding energy of the C 1s peak calibrated at 284.5 eV was utilized as a charge reference. Figure 4A displays the complete survey scan spectra of the CF/CuO/ZIFs (Co_x_·Zn_1−x_O)@BMOF(Ni–Mn) electrode, revealing the presence of key components including O, Mn, Ni, Zn, Co, and Cu. Deconvoluting the O 1s yielded three probable Gaussian fits. As can be seen in Fig. 4B, the peak recorded at 529.3 eV corresponded to metal–oxygen bonding, the peak referred to the lattice oxygen was observed at 533.1 eV, the peak positioned at 534.4 assigned to the presence of adsorbed oxygen^39^. In the high-resolution Mn 2p spectrum of the CF/CuO/ZIFs (Co_x_·Zn_1−x_O)@BMOF(Ni–Mn) electrode, the doublet peaks corresponding to Mn 2p_3/2_ and Mn 2p_1/2_ were positioned at 643.62 and 654.15 eV, respectively (shown in Fig. 4C). Three peaks were observed at 641.8, 644.90, and 648.3 eV in the deconvoluted Mn 2p_3/2_ spectrum indicating that the Mn^2+^ and Mn^3+^ (oxidation states of Mn in binary MOF structure) and satellite peak, respectively. The deconvoluted Mn 2p_1/2_ peak displays the presence of a primary peak located at 654.15 eV corresponding to Mn^3+^^40,41^. Figure 4D exhibits the XPS measurement of Ni 2p spectrum for the electrode. The lower peak intensity seen at 854.6 eV refers to Ni 2p_3/2_. The aforementioned characteristics are fundamentally corresponded to the value of the co-existence of Ni^2+^ and Ni^3+^. Another peak position in Ni 2p_3/2_ observed at 859.7 refers to the satellite peak. The deconvoluted Ni 2p exhibits two peaks at high peak intensity, with centers at approximately 875.2 and 877.90 eV, which are attributed to the Ni 2p_1/2_ and satellite peaks, respectively. Mn and Ni both have valence states of + 2 and + 3 at the same time^42,43^. The presence of different valence states of metal elements will enhance the catalytic activity active site and strengthen the redox performance of the MOF, hence providing considerable benefits to the energy storage performance. The presence of Ni and Mn embedded in the MOF structure during the final synthesis step is verified in Fig. 4C,D. As can be seen in Fig. 4E, the Co 2p spectra represent two peaks in the curve at 782.6 and 797.5 eV attributed to Co 2p_3/2_ and Co 2p_1/2_ states, respectively. The Co 2p_3/2_ spectrum was deconvoluted into two fitted peaks at 781.20 eV and 783.41 eV, which can be attributed to the Co^2+^ and Co^3+^ oxidation states, respectively. On the other hand, The Co 2p_1/2_ was deconvoluted into two fitted peaks at 796.41 eV and 797.61 eV, indicating the presence of Co^2+^ and Co^3+^ oxidation states, respectively. The oxidation states Co 2p_3/2_ and Co 2p_1/2_ are confirmed by the presence of two satellite peaks at 785.3 eV and 805.71 eV, respectively^44^. As shown in Fig. 4F, the high-resolution Zn 2p spectra exhibits two distinct peaks at around 1022.4 and 1044.1 eV, which correspond to the Zn 2p_3/2_ and Zn 2p_1/2_ oxidation state, respectively. The presence of these peaks can be ascribed to the Zn^2+^ oxidation state. Therefore, it can be inferred that the doped ZnO polycrystal contains the Co phase. Figure 4G displays the Cu 2p spectrum with a prominent level of detail. The two primary peaks, located at around 935.4 eV and 948.2 eV, correspond to the Cu 2p_3/2_ and Cu 2p_1/2_ peaks, respectively. The Cu 2p_3/2_ and Cu 2p_1/2_ states are further analyzed, revealing the existence of both 1+ and 2+ oxidation states^45,46^. The conspicuous satellite peak suggests that the predominant oxidation state of copper was Cu^2+^. The inclusion of metallics in the final electrode serves as active sites for enhanced Faradaic redox reactions, resulting in superior capacitive performance. This finding provides more evidence that binary MOF doping can increase the active site in CF/CuO/ZIFs (Co_x_·Zn_1−x_O)@BMOF(Ni–Mn), which aligns well with the observations made through the FESEM image.

### Electrochemical performance

**Figure 5:**
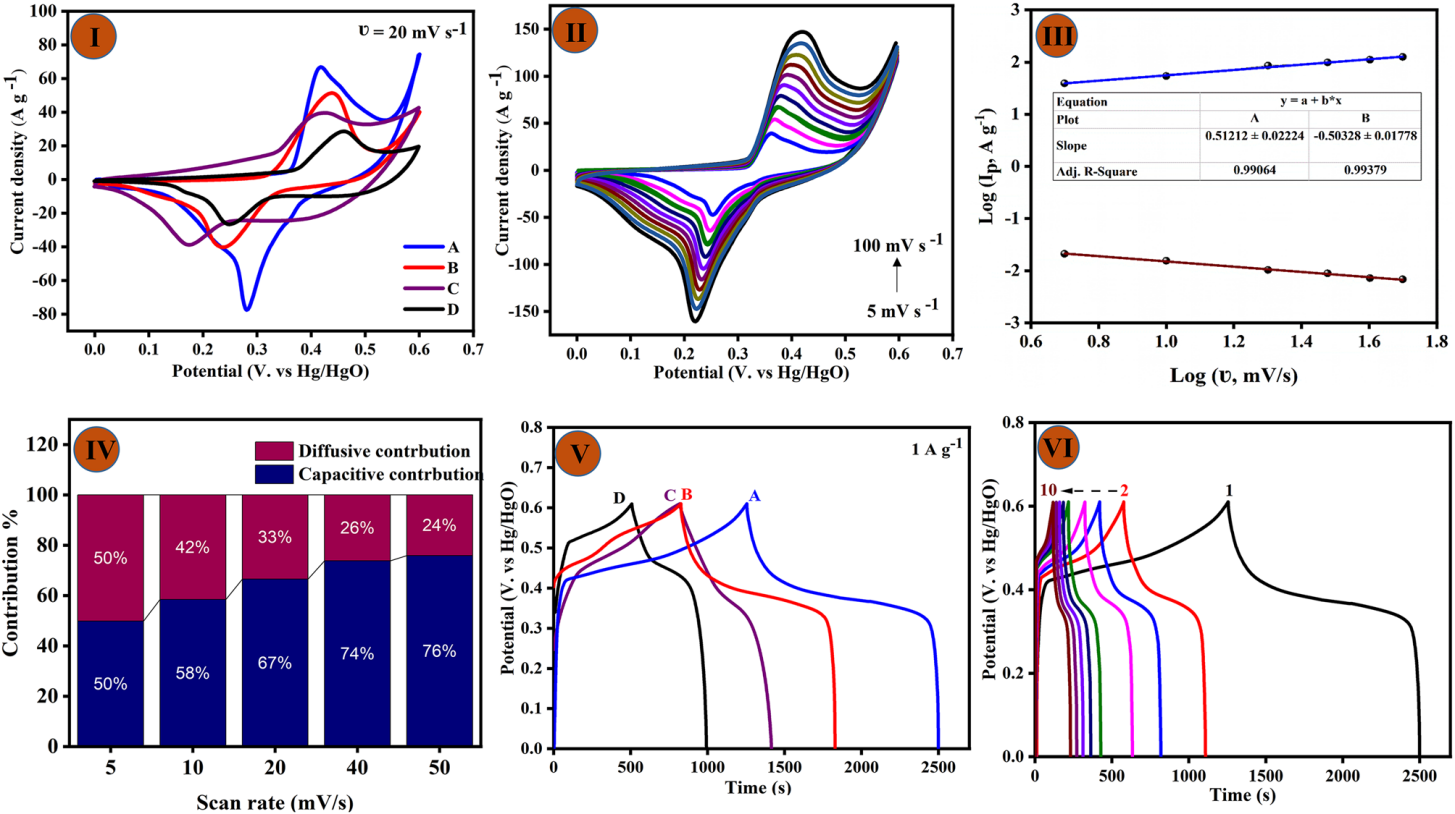
(**I**) CVs of the (**A**) CF/CuO/ZIFs (Co_x_·Zn_1−x_O)@BMOF(Ni–Mn), (**B**) CF/CuO/ZIFs (Co_x_·Zn_1−x_O), (**C**) CuO/CF and (**D**) Cu (OH)_2_/CF (at the scan rate 20 mV s^−1^); (**II**) CV curves of the CF/CuO/ZIFs (Co_x_·Zn_1−x_O)@BMOF(Ni–Mn) at different scan rate (5–100 mV s^−1^); (**III**) log I vs. log ʋ for electrode materials, (**IV**) percent of contribution at different scan rate. (**V**) GCD profiles of (**A**) CF/CuO/ZIFs (Co_x_·Zn_1−x_O)@BMOF(Ni–Mn), (**B**) CF/CuO/ZIFs (Co_x_·Zn_1−x_O), (C) CuO/CF, and (**D**) Cu (OH)_2_/CF at 1 A g^−1^, (**VI**) GCD profiles of the CF/CuO/ZIFs (Co_x_·Zn_1−x_O)@BMOF(Ni–Mn) electrode at various current densities (1–10 A g^−1^).

To assess the efficacy of CF/Cu(OH)_2_, CF/CuO, CF/CuO/ZIFs (Co_x_·Zn_1−x_O), and CF/CuO/ZIFs (Co_x_·Zn_1−x_O)@BMOF(Ni–Mn) electrode as energy storage materials, we conducted investigations using cyclic voltammetry (CV), galvanostatic charge–discharge (GCD), and electrochemical impedance spectroscopy (EIS) techniques. The studies were conducted utilizing a 3-electrode setup in an aqueous electrolyte containing 3.0 M KOH. As can be seen in Fig. 5I, the fabricated electrode materials were evaluated by CV analysis at a scan rate of 20mV/s. The purpose was to examine the potential pseudo-faradaic contributions throughout the potential range of 0.0–6.0 V vs. Hg/HgO at room temperature. Nevertheless, a quasi-reversible peak was detected at approximately 0.46 V vs. Hg/HgO for CF/Cu(OH)_2_, indicating the oxidation of copper species. Additionally, a reduction peak was identified at around 0.27 V vs. Hg/HgO during the negative scan. Typical CV curves reflecting the nature of the electrochemical behavior of CF/CuO in a potential range of 0.0 to 0.6 V vs. Hg/HgO are shown. The CF/CuO/ ZIFs (Co_x_·Zn_1−x_O) electrodes showed a board redox peak, which confirms their faradaic behavior that could correspond to the reversible redox of Zn and Co, with an additional redox peak of Cu^1+^/Cu^2+^ for CuO substrate. These results illustrate the ratio/proportion of the integral area, suggesting that the combination CF/CuO/ZIFs (Co_x_·Zn_1−x_O) has a greater integrated area compared to the other electrode materials. Furthermore, the addition of binary metal MOF in the structure resulted in a substantial increase in the area under the CV curve. This suggests that the inclusion of Mn and Ni into the MOF structure enhances the capacitive performance of the electrodes. The entire reaction can be attributed to the combination of M (representing Cu/Zn/Co/Ni/Mn) and OH anion derived from the KOH electrolyte.

$$\text{MO }+ {\text{OH}}^{-} \leftrightarrow \text{ MOOH }+ {\text{e}}^{-}.$$

Among all CVs records best specific capacity related to CF/CuO/ZIFs (Co_x_·Zn_1−x_O)@BMOF(Ni–Mn) due to its high surface area and good crystallinity as it was confirmed via FE-SEM images.

As shown in Fig. 5II, the CV curves of CF/CuO/ZIFs (Co_x_·Zn_1−x_O)@BMOF(Ni–Mn) are also studied at various scan rates. As the scan rate increased from 5 to 100 mV s^−1^, the current density area of the redox peak became larger. The almost symmetrical CV curves for CF/CuO/ZIFs (Co_x_·Zn_1−x_O)@BMOF(Ni–Mn) suggest that the electrode materials were advantageous for fast redox reactions, and exceptional rate capability. Furthermore, the anodic and cathodic peaks exhibited a shift towards positive and negative potentials, respectively. This shift can be attributed to the polarization of the electrode materials as well as the fast transport of electrons and ions. On the other hand, the scan rate increase results in decreased electrolyte interaction with the electrochemically active species, which consequently hinders reaction kinetics.

Furthermore, the analysis of both Faradaic and non-Faradaic processes can be conducted by plotting the logarithm of the peak current (anodic and cathodic) against the logarithm of the scan rate (Log I = b log(ʋ) + log(a)). The scan rate is indicated by ʋ, current (i), whereas a and b are constants. The value of b can be ascertained from the slope of the aforementioned plot. On the other hand, the charge storage characteristics of the electrode materials are determined by the value of b. When the value of b is 1, the process is solely capacitive, namely an electric double-layer capacitor (EDLC). Conversely, when the value of b is 0.5, the process can be categorized as a diffusion-controlled faradaic reaction. The b values were determined for CF/CuO/ZIFs (Co_x_·Zn_1−x_O)@BMOF(Ni–Mn), yielding a cathodic peak value of 0.512 and an anodic peak value of − 0.503 (As shown in Fig. 5III,IV. These findings indicate that the electrode’s charge storage mechanism was mostly influenced by a diffusion-controlled process. As a result, the electrode displayed a behavior like that of a battery type. This study provides more evidence confirming the presence of two distinct charge storage mechanisms in these electrochemical processes. The methodology developed by Dunn was employed to categorize the entire capacity into two separate components: capacitive behavior (capacitive contribution (k_1_ ν)) and diffusion-controlled activity (k_2_ ν^1/2^) at a fixed potential (V). The k_1_ and k_2_ values can be determined by following the equation and analyzing the linear correlation between i/v^1/2^ and v^1/2^.

**Table:** 

Equation	Deform equation
i(V) = k_1_ ν + k_2_ ν^1/2^	$\frac{i}{{\upnu }^{1/2}}$ = k_1_ ν^1/2^ + k_2_

The CF/CuO/ZIFs (Co_x_·Zn_1−x_O)@BMOF(Ni–Mn) exhibits a leading contribution from diffusion control over kinetic control, suggesting that the response is mostly controlled by diffusion.

Figure 5V illustrates the comparison of GCD curves for several electrode materials at a current density of 1 A g^−1^ within the potential window range of 0.0–0.6 V vs. Hg/HgO. Among all electrode materials, The CF/CuO@Zn/Co-MOF(Ni–Mn) displays greater specific capacity if compared with other electrode materials at the same current density. The specific capacity of CF/Cu(OH)_2_, CF/CuO, CF/CuO/ZIFs (Co_x_·Zn_1−x_O), and CF/CuO/ZIFs (Co_x_·Zn_1−x_O)@BMOF(Ni–Mn) electrode was 1249.99, 600.66, 355.74, and 294.37 C g^−1^ at 1 A g^−1^. The CF/CuO/ZIFs (Co_x_·Zn_1−x_O)@BMOF(Ni–Mn) materials electrode exhibits excellent capacity performance at different current densities, with specific capacity values of 1249.99, 1140.25, 1080.12, 1000.76, 950.61, 900.37, 889.79, 880.91, 810.28 and 800.19 C g^−1^ at 1.0, 2.0, 3.0, 4.0, 5.0, 6.0, 7.0, 8.0,9.0 and 10 A g^−1^, respectively (shown in Fig. 5VI).

**Figure 6:**
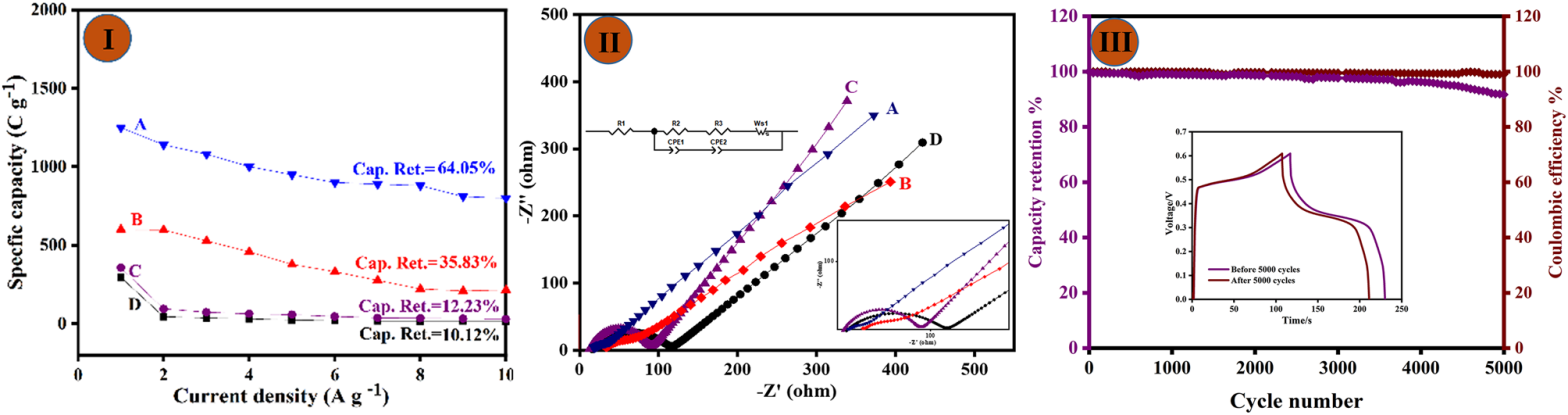
(**I**) Specifics capacity of (**A**) CF/CuO/ZIFs (Co_x_·Zn_1−x_O)@BMOF(Ni–Mn), (**B**) CF/CuO/ZIFs (Co_x_·Zn_1−x_O), (**C**) CuO/CF, and (**D**) Cu(OH)_2_/CF at different current density (1 to 10 A g^−1^). (**II**) The electrochemical impedance spectroscopy (EIS) spectrum of the following electrodes was obtained: (**A**) CF/CuO/ZIFs (Co_x_·Zn_1−x_O)@BMOF(Ni–Mn), (**B**) CF/CuO/ZIFs (Co_x_·Zn_1−x_O), (**C**) CuO/CF, and (**D**) Cu(OH)_2_/CF. The spectrum was analyzed using an equivalent circuit. (**III**) Cyclic performance and coulombic efficiency of the CF/CuO/ZIFs (Co_x_·Zn_1−x_O)@BMOF(Ni–Mn) at the current density 10 A g^−1^ (the inset shows the GCD of the electrode before and after 5000 cycles).

As can be seen in Fig. 6I, the CF/Cu(OH)_2_ and CF/CuO electrodes show a limited capacity to maintain their performance at different current densities, with a rate capability of 10.12 and 12.23 respectively, primarily due to the absence of transition metals in electrode materials. Comparatively, the CF/CuO/ZIFs(Co_x_·Zn_1−x_O) and CF/CuO/ZIFs(Co_x_·Zn_1−x_O)@BMOF(Ni–Mn) both show the enhanced rate capabilities of 35.83% and 64.05% respectively. This result can be ascribed to the CF/CuO/ZIFs(Cox·Zn_1−x_O) substrate synergetic effect and the electroactivity (Ni/Mn) in the binary MOF and the rate capabilities of the electrode material were increased due to the integration of Ni/Mn to form nanoflower-like MOF as shown in FESEM images.

To assess the conductivity and rate of charge transfer of the fabricated electrodes, Electrochemical Impedance Spectroscopy (EIS) was performed. The frequency range used was 0.1–104 kHz (shown in Fig. 6II). The data obtained using EIS is presented in the form of a Nyquist plot. The resistance is attributed to three factors: the electrolyte’s resistance, the active material’s resistance in the electrode, and the resistance at the interface between the current collector and the active material. Each data point on the Nyquist plot represents an impedance at a distinct frequency. The analogous series resistance (Rs) or solution resistance is the point where the real axis intersects in the high-frequency zone. On the other hand, a semicircle observed at high frequencies signifies a direct relationship between the charge transfer resistance (Rct) and the diameter of the semicircle. The values of Rct and Rs for each electrode material are provided in Table 1. It can be contended that the resistance values (Rs) stem from a shared point and stay uniform across all electrodes.

**Table 1:** Solution and charge transfer resistance (Rs and Rct) of electrode materials.

Electrode materials	Solution resistance Rs (Ω)	Charge transfer resistance Rct (Ω)
CF/ Cu (OH)_2_	7.1	58.2
CF/CuO	7.8	44.25
CF/CuO/ZIFs (Co_x_·Zn_1−x_O)	17.2	28.3
CF/CuO/ZIFs (Co_x_·Zn_1−x_O)@BMOF(Ni–Mn)	8.5	19.2

It can be concluded that the addition of Ni–Mn-based MOF to CF/CuO/ZIFs (Co_x_·Zn_1−x_O) led to a significant decrease in its resistance, due to the improvement in its conductivity, hence strengthening its electrochemical performance. These EIS findings align well with the results obtained from GCD and CV tests.

Figure 6III demonstrates the Stability test of the CF/CuO/ZIFs (Co_x_·Zn_1−x_O)@BMOF(Ni–Mn) electrode, which underwent 5000 GCD cycles at a current density of 10 A g^−1^. The Ni–Mn-based MOF introduced to CF/CuO/ZIFs (Co_x_·Zn_1−x_O) was well maintained and preserved even after 5000 cycles. In addition, the electrode materials exhibited exceptional reversibility, as evidenced by the attainment of a 98.7% coulombic efficiency after 5000 cycles. The cycling performance of CF/CuO/ZIFs (Co_x_·Zn_1−x_O)@BMOF(Ni–Mn) was evaluated, revealing a cycling stability of 91.74% after 5000 cycles. Hence, it can be inferred that the addition of Ni–Mn-based MOF is the optimal choice for augmenting the electrochemical reactivity of CF/CuO/ ZIFs (Co_x_·Zn_1−x_O), making it a distinctive alternative among electrode materials for supercapacitors.

### Asymmetric supercapacitor (ASC)

**Figure 7:**
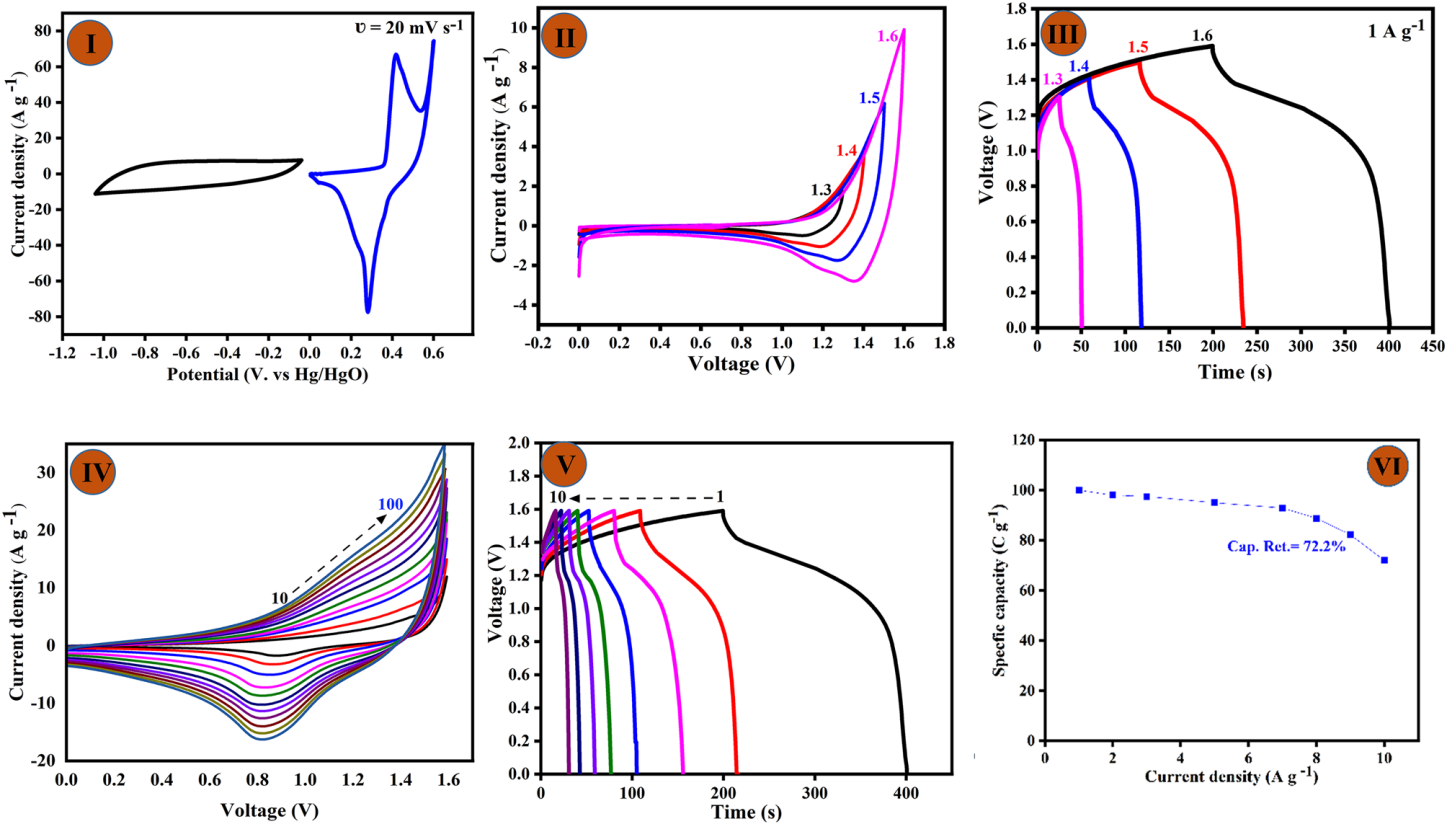
Electrochemical study of asymmetric supercapacitor fabricated using CF/CuO/ZIFs (Co_x_·Zn_1−x_O)@BMOF(Ni–Mn) and AC/CF electrode: (**I**) CV curves showing potential windows for anode and cathode. (**II**) CV profile for different potential windows. (**III**) GCD curves at constant current densities and different potential windows. (**IV**) CV profile at different scan rates (10 to 100 mV s^−1^). (**V**) GCD plot at different current densities (1 to 10 A g^−1^). (**VI**) Specifics capacity of CF/CuO/ZIFs (Co_x_·Zn_1−x_O)@BMOF(Ni–Mn)//AC device at different current densities (1 to 10 A g^−1^).

CF/CuO/ZIFs (Co_x_·Zn_1−x_O)@BMOF(Ni–Mn) electrode was used as the positive electrode for the asymmetric supercapacitor (ASC) device because it performed so well as a supercapacitor. The energy density of the ASC device can be increased by combining electrode materials with different chemical properties. To achieve this as a high-capacity supercapacitor, CF/CuO/ZIFs (Co_x_·Zn_1−x_O)@BMOF(Ni–Mn) material can be combined with the EDLC-type material (such as activated carbon (AC)). As can be seen in Fig. 7I, the CV plots of the CF/CuO/ZIFs (Co_x_·Zn_1−x_O)@BMOF(Ni–Mn) electrode (ranging from 0.0 to 0.6 V) and AC electrode (ranging from − 1.0 to 0.0 V) materials indicate that the high-capacity supercapacitor device can function across a broad potential range of 1.6 V. The CV plots of the negative electrode, consisting of nickel foam coated with AC, display a nearly rectangular shape within the stable potential range, indicating the characteristic behavior of EDLC-type material. The CV profile of the positive electrode (CF/CuO/ZIFs (Co_x_·Zn_1−x_O)@BMOF(Ni–Mn)) exhibits two separate redox peaks, which is indicative of the Faradaic-pseudo capacitance behavior. To assess the stable potential window of the as-assembled cell, a set of CV curves was obtained, as depicted in Fig. 7II. These curves reveal a consistent voltage range of 0.0 to 1.6 V, indicating stability. Expanding the potential window further will cause phenomena such as oxygen evolution reaction, water splitting, and electrolyte decomposition, resulting in a reduction in capacity and disruption of the system’s performance. The remarkable performance of the fabricated device, which is attributed to the combined effect of the EDLC-type material (AC) and the faradaic response of CF/CuO/ZIFs (Co_x_·Zn_1−x_O)@BMOF(Ni–Mn) can be observed in Fig. 7IV. This CV profile was recorded at a various scan rate (10 to 100 mV s^−1^) for CF/CuO/ZIFs (Co_x_·Zn_1−x_O)@BMOF(Ni–Mn)//AC device exhibit high performance capacitive behavior. The CV profile maintained its shape despite the higher scan rates, suggesting that the ACS device has a good-rate capability, excellent reversibility, fast charge/discharge properties, and rapid electron transfer kinetics.

**Figure 8:**
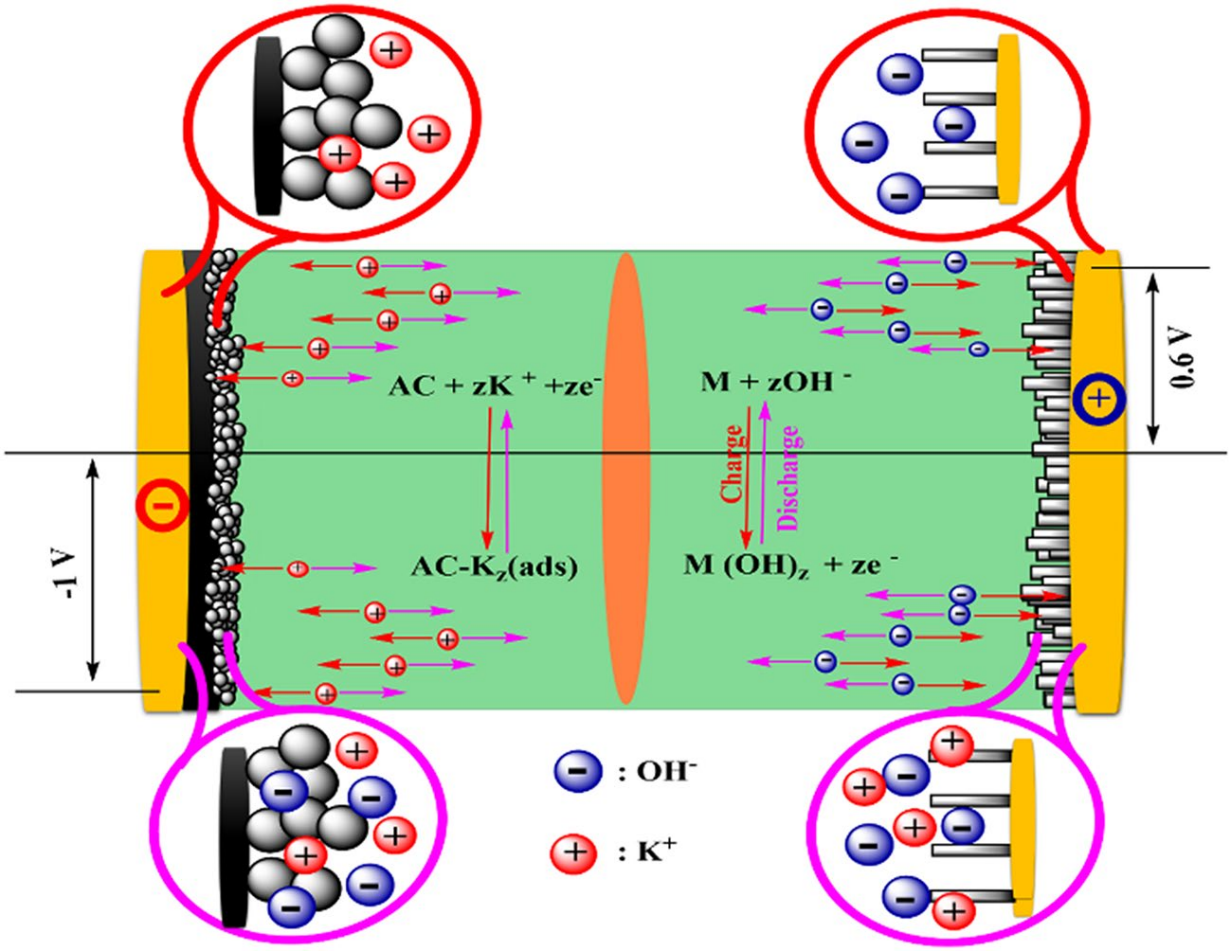
Proposed energy storage mechanism through schematic illustration for CF/CuO/ZIFs (Co_x_·Zn_1−x_O)@BMOF(Ni–Mn)//AC device.

As shown in Fig. 7III, the GCD curves of the ACS device were recorded at current densities of 1 A g^−1^ in the potential window range of 0.0 to 1.6 V. The GCD curves closely align with the CV curves, and the observed asymmetrical GCD curve for CF/CuO/ZIFs (Co_x_·Zn_1−x_O)@BMOF(Ni–Mn)//AC indicates battery-like behavior. The specific capacity of the as-fabricated ACS device was estimated by evaluating the GCD curves recorded at various current densities (1 to 10 A g^−1^) that have similar shapes that indicate the possibility of electrochemical reversibility (shown in Fig. 7V). The ACS device yielded a maximum specific capacity of 100.47 C g^−1^ at a current density of 1 A g^−1^. Figure 7VI displays the specific capacity values achieved at various current densities. The specific capacity of CF/CuO/ZIFs (Co_x_·Zn_1−x_O)@BMOF(Ni–Mn)//AC device at 1.0, 2.0, 3.0, 5.0, 7.0, 8.0, 9.0, and 10 A g^−1^ are 100.47, 98.14, 97.48, 94.99, 92.89, 88.6, 72.24 and 72.17 C g^−1^, respectively. Hence, the specific capacity retains 72.2% of its initial value when the current density is raised to 10.0 A g^−1^. The durability and coulombic efficiency of the ACS device in its developed state is also a crucial component in evaluating the overall performance of practical applications. The proposed energy storage mechanism is depicted in Fig. 8. The CF/CuO/ZIFs (Co_x_·Zn_1−x_O)@BMOF(Ni–Mn) electrode facilitates the oxidation reaction in the KOH electrolyte by using OH^−^ ions during the charging phase. During discharge, there is an electrochemical reduction.

**Figure 9:**
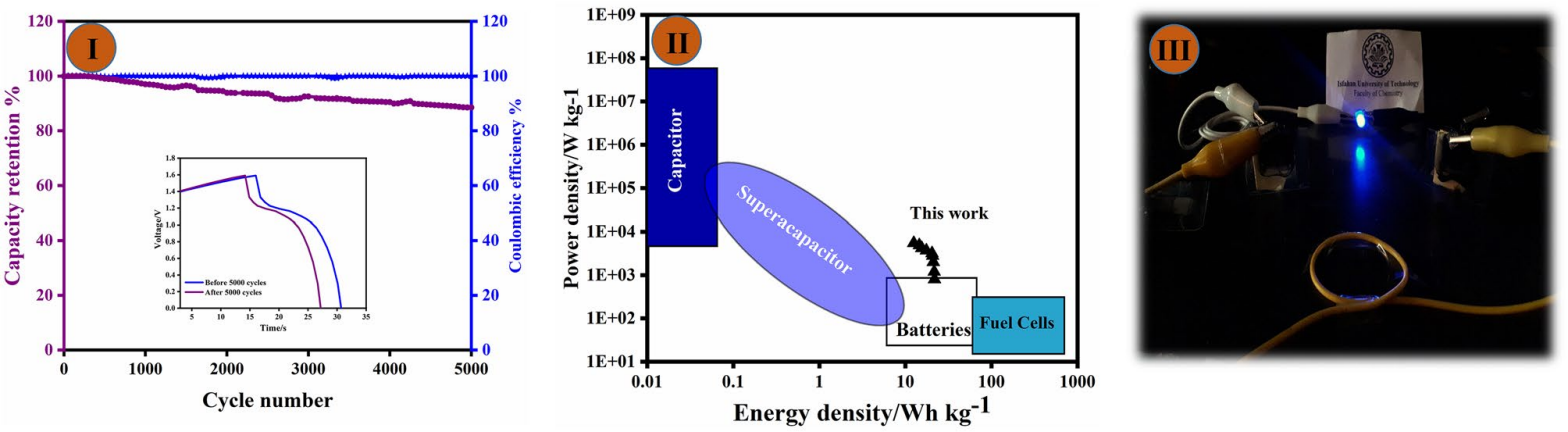
(**I**) Cyclic stability and coulombic efficiency test for 5000 cycles of charge/discharge. (**II**) The Ragone plot for energy and powder densities of the CF/CuO/ZIFs (Co_x_·Zn_1−x_O)@BMOF(Ni–Mn)//AC device along with a comparison of previously reported similar works. (**III**) The image of the LED being powered by the CF/CuO/ZIFs (Co_x_·Zn_1−x_O)@BMOF(Ni–Mn)//AC device.

As shown in Fig. 9I, the ACS device underwent 5000 charge–discharge cycles at a discharge rate of 10 A g^−1^. The cell maintained 88.5% of its initial specific capacity, demonstrating the strong and durable quality of the electrode material used. The first and final cycles are displayed in the inset of Fig. 9I. On the other hand, the ACS device had a columbic efficiency of 88.52% after 5000 cycles of charge–discharge. Energy density and power density are crucial factors in assessing the performance of ASC devices. The experimental findings indicate that the CF/CuO@Zn/Co-MOF(Ni–Mn)//AC device possesses an energy density of 21.77 Wh kg^−1^ and a power density of 799.19 Wh kg^−1^.

Notably, the energy density and power density of the ASC device were compared to those of some of the previous devices based on the MOF and multi-metallic electrode with the same transition metal (shown in Fig. 9II). Figure 9III demonstrates the effective application of the ASC device, where two ACS devices were connected in series, to power blue light-emitting diodes (LEDs). This demonstrates the practical use of CF/CuO/ZIFs (Co_x_·Zn_1−x_O)@BMOF(Ni–Mn) electrode materials. Finally, an in-depth analysis of the AC asymmetric supercapacitor was conducted in comparison to other electrochemical pseudo capacitors (shown in Table 2).

**Table 2:** An analysis of various AC asymmetric supercapacitors.

Electrode materials	Symmetric devices (3E)		Asymmetric devices (2E)			Number of cycles	Ref.
	Electrolyte	Specific capacity	Energy density (Wh kg^−1^)	Power density (W kg^−1^)	Capacitance retention (%)		
MnCoNi-LDH@CoNi-LDH	6 M KOH	2126.7F g^−1^ (0.5 A g^−1^)	51.4	7500	104.1	10,000	^47^
Ni2S@C/CNTs	2 M KOH	1572F g^−1^ (0.5 A g^−1^)	21.6	–	94.8	10,000	^48^
Ni–Mn LDH/Co_3_O_4_/CP	1 M KOH	1327 F g^−1^ (1 A g^−1^)	35.847	449.99	84.8	16,000	^49^
Ni/Co MOF	2 M KOH	339.3 C g^−1^ (1 A g^−1^)	23.44	350	96	10,000	^50^
NiCo_2_O_4_- MOF	3 M KOH	531 Fg^−1^ (1 A g^−1^)	9.4	82	96	1000	^51^
KCu7S4@NiMn LDH	1 M LiOH	733.8 F g^−1^	15.9	9.4	84.8	16,000	^52^
CF/CuO/ZIFs (Co_x_·Zn_1−x_O)@BMOF(Ni–Mn)	1M KOH	1249.99 C g^−1^ (1 A g^−1^)	21.77	799	88.52	5000	This work

## Conclusions

In summary, CF/CuO/ZIFs (Co_x_·Zn_1−x_O)@BMOF(Ni–Mn) electrodes consisting of decorated flower-like BMOF and ZIFs (Co_x_·Zn_1−x_O) over nanowire CuO/CF nanostructures were successfully synthesized by simple hydrothermal technique. The morphology and synthesis stage has a significant impact on the performance of the fabricated electrodes in terms of supercapacitor performance. The binary MOF decorated over CF/CuO/ZIFs (Co_x_·Zn_1−x_O) structure, increases the electrochemically active surface area, facilitating faradaic reactions and magnifying the synergistic effects of pseudo-electrode materials. The specific capacity at current density (1 A g^−1^) was improved from 600.6 C g^−1^ (CF/CuO/ZIFs (Co_x_·Zn_1−x_O)) to 1249.99 C g^−1^ (CF/CuO/ZIFs (Co_x_·Zn_1−x_O)@BMOF(Ni–Mn)). The Cyclic performance and coulombic efficiency were 91.74% and 98.7%, respectively after 5000 GCD cycles. The assembled asymmetric device displays a retention in capacity of 88.52% after 5000 GCD cycles and delivered a high energy density of 21.77 Wh kg^−1^ and a power density of 799.19 Wh kg^−1^. The noteworthy results indicated that combining and designing binary MOF and transition metal oxide on a CuO substrate is a potential approach to enhance the efficiency of electrodes in energy storage applications.

## Experimental

### Chemicals and apparatus

The chemical materials utilized in this work were employed without additional purification. Analytical grade potassium hydroxide (KOH), ethanol (C_2_H_6_O), and hexamethylenetetramine ((CH_2_)_6_N_4_), activated carbon (AC), ammonium persulfate ((NH_4_)_2_S_2_O_8_, APS), sodium hydroxide (NaOH), nickel dichloride hexahydrate (NiCl_2_·6H_2_O), manganese chloride (MnCl_2_·4H_2_O), 2-methylimidazole (C_4_H_6_N_2_), cobalt(II) nitrate hexahydrate (Co(NO_3_)_2_·6H_2_O), zinc nitrate hexahydrate (Zn(NO_3_)_2_·6H_2_O), and methanol (CH_3_OH) were bought from Sigma–Aldrich (St Louis, MO). A copper foam was bought from Redox Kala Company (Tehran, Iran). The experiments were conducted using deionized water.

### Apparatus

The surface oxidation and composition of CF/CuO/ZIFs (Co_x_·Zn_1−x_O)@BMOF(Ni–Mn) were analyzed using X-ray photoelectron spectroscopy (XPS) with the Bes Tec 8025 instrument. Furthermore, the shape and elemental composition of the fabricated electrodes were analyzed using field-emission scanning electron microscopy (FE-SEM, a TESCAN MIRA3 LMU instrument (Czech Republic)) in conjunction with energy dispersive X-ray (EDX) analysis. The fabricated materials conducted electrochemical testing utilizing a potentiostat/galvanostat Biologic SP300 controlled by software. The electrochemical behavior was recorded in a 3.0 mol L^−1^ (KOH) solution, which acted as the electrolyte. The experimental configuration comprised a three-electrode design, featuring a reference electrode composed of HgO/Hg and a counter electrode composed of Pt wire.

### Electrodes fabrication

#### Synthesis of CuO nanowire on CF

The synthesis of CuO substrate was conducted by the methodology outlined in the prior literature^53^. The CuO layer was synthesized using a hydrothermal method. The Copper foam (CF) substrate was initially sectioned into small pieces (1 × 1 cm with a thickness of 0.1 cm), and then thoroughly cleaned using 1.0 mol L^−1^ HCl, ethanol, and acetone in a sonicated for 30 min. This cleaning process effectively removes contaminants and oxide layers from the substrate. The CF that had been prepared was submerged in a solution consisting of an APS solution (0.13 mol L^−1^), and NaOH (2.5 mol L^−1^) in 20 mL deionized water for 20 min at room temperature. The resulting product was rinsed with distilled water and ethanol. The laundered material was subjected to a drying process at a temperature of 60 °C overnight. The Cu(OH)_2_ layer has an estimated weight of around 2.41 mg cm^−2^. The Cu(OH)_2_ layer underwent a heating process in an oven with a nitrogen gas environment. The temperature was kept at 180 °C for 2 h. The estimated weight of the dark brown CuO nanowires is approximately 1.48 mg cm^−2^.

#### Synthesis of CF/CuO/ZIFs (Co_x_·Zn_1−x_O)

To execute a standard synthesis with the approach that has been documented^54^, a solution was prepared by dissolving Co(NO_3_)_2_∙6H_2_O (0.16 mmol L^−1^) and Zn(NO_3_)_2_∙6H_2_O (0.08 mmol L^−1^) in 30 mL of methanol, resulting in a translucent solution. Subsequently, a solution of 2-methylimidazole (12 mmol L^−1^) in 20 mL of methanol was added. After being thoroughly homogenized through continuous agitation for 10 min, the solution and CF/CuO electrode were thereafter placed in an autoclave and subjected to incubation at a temperature of 100 °C for 12 h. Following the drying process, the electrode was subjected to calcination by placing it in a tube furnace at a temperature of 350 °C for 3 h. The electrode's weight was calculated to be around 1.86 mg cm^−2^.

#### Synthesis of CF/CuO/ZIFs (Co_x_·Zn_1−x_O)@BMOF(Ni–Mn)

The BMOF with hexamethylenetetramine linker was synthesized following the methodology described in the previous literature^55^. To generate a uniform solution, 0.8 mmol of NiCl_2_·6H_2_O, 3.8 mmol of MnCl_2_·4H_2_O, and 5.4 mmol of hexamethylene triamine were dissolved in 50 mL of deionized water using a sonicate bath. Subsequently, the solution was transferred into a 100 mL Teflon container, followed by the placement of the CF/CuO/ZIFs (Co_x_·Zn_1−x_O) electrode within the Teflon container solution. The entire setup was then subjected to autoclaving at a temperature of 85 °C for 6 h. Following the synthesis process, the sample was taken out of the autoclave and cooled gradually to room temperature. Finally, it was placed in an oven at a temperature of 60 °C for 6 h. An amount of 2.33 mg cm^−2^ of CuO/ZIFs (Co_x_·Zn_1−x_O)@BMOF(Ni–Mn) was deposited onto the CF surface.

### Assembly of asymmetrical supercapacitors

Assembly of the ASC devices involved the utilization of activated carbon (AC) as the negative electrode, and the CF/CuO/ZIFs (Co_x_·Zn_1−x_O)@BMOF(Ni–Mn) (prepared as such in a three-electrode system) as the positive electrode. The asymmetric supercapacitor device utilizes a Whatman paper to prevent a short circuit between the positive and negative electrodes. To optimize the performance and ensure a broad potential window in the battery-like SCs system, the charge must be in equilibrium between the positive (q^+^) and negative (q^−^) electrodes, with the ideal mass ratio (m^+^/m^−^) calculated using the subsequent equation:

$${m}^{+}/{m}^{-}={C}^{-}{V}^{-}/{C}^{+}{V}^{+},$$

according to the preceding equation, the ideal mass ratio for the as-fabricated asymmetric supercapacitor is almost 0.157.

## Data Availability

The datasets supporting the conclusions of this article are included within the article.
